# Crystal and geometry-optimized structure, and Hirshfeld surface analysis of 1-(2-bromo­eth­yl)indoline-2,3-dione

**DOI:** 10.1107/S2056989016015760

**Published:** 2016-10-11

**Authors:** N. Sharmila, T. V. Sundar, G. Sathish, P. Venkatesan

**Affiliations:** a Postgraduate and Research Department of Physics, National College (Autonomous), Tiruchirappalli 620 001, Tamilnadu, India; b School of Chemistry, Bharathidasan University, Tiruchirappalli 620 024, Tamilnadu, India; c Laboratorio de Políimeros, Centro de Química Instituto de Ciencias, Benemérita Universidad Autónoma de Puebla (BUAP), Complejo de Ciencias, ICUAP, Edif. 103H, 22 Sur y San Claudio, C.P. 72570 Puebla, Puebla, Mexico

**Keywords:** crystal structure, isatin, C—H⋯O hydrogen bonds

## Abstract

In the title compound, C_10_H_8_BrNO_2_, the isatin (1*H*-indole-2,3-dione) moiety is nearly planar (r.m.s. deviation = 0.026 Å). In the crystal, mol­ecules are linked by C—H⋯O hydrogen bonds, forming layers parallel to the *ab* plane, and enclosing *R*_4_^4^(24) loops. There are a low percentage (19.3%) of inter­molecular H⋯H contacts in the structure, as estimated by the analysis of Hirshfeld surfaces. This could be due to the presence of the Br atom, present in the bromo­ethyl­ene group, which makes *ca* 18.7% Br⋯H contacts.

## Abstract 2

In the title compound, the isatin (1*H*-indole-2,3-dione) moiety is almost planar (r.m.s. deviation = 0.026 Å). In the crystal, mol­ecules are linked by C—H⋯O hydrogen bonds, forming layers parallel to the *ab* plane, and enclosing 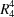
(24) loops.

## Chemical context   

Isatin (1*H*-indole-2,3-dione) is an endogenous compound that has been identified in humans and possesses a wide range of biological activities, such as anxiogenic and sedative activities. It serves as a synthetically useful substrate which can be used to prepare a broad range of heterocyclic compounds, including mol­ecules of pharmacological significance (Bekircan & Bektas, 2008 ▸). A variety of biological activities are associated with isatin, including central nervous system (CNS) activities (Raj, 2012 ▸). As part of our inter­est in the identification of bioactive compounds, we report herein on the synthesis, the crystal structure, and the geometry optimization and Hirshfeld surface analysis of the title isatin derivative, (I).
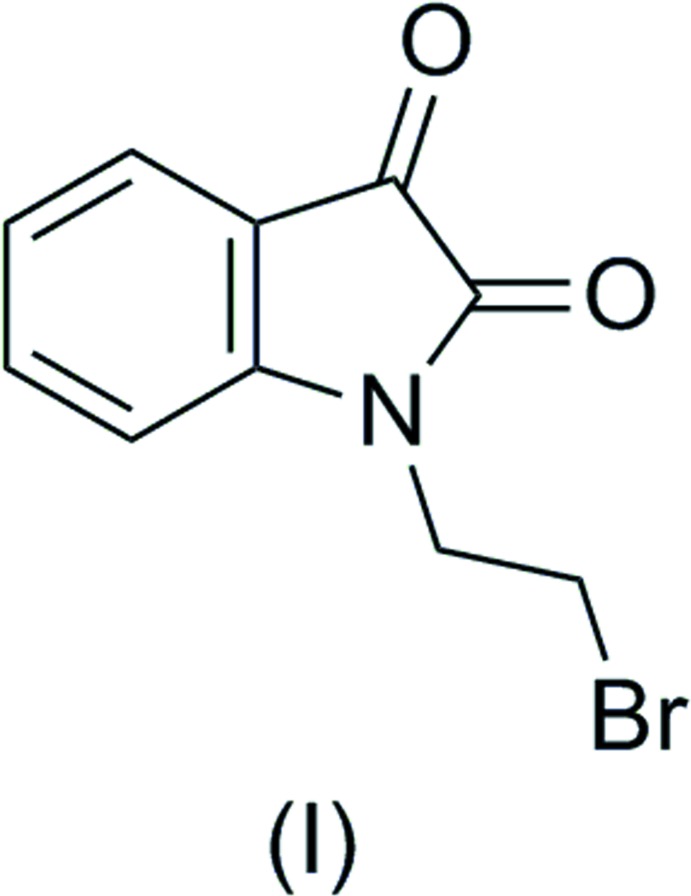


## Structural commentary   

**Figure 1:**
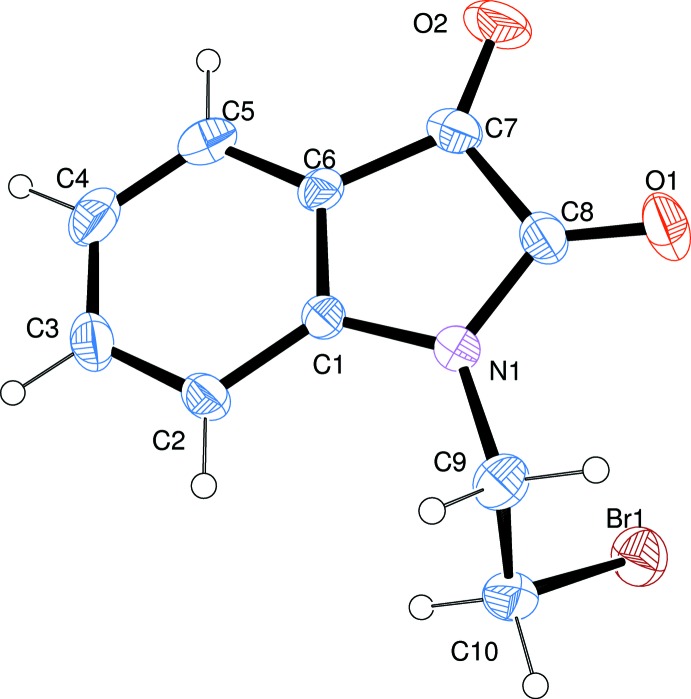
The mol­ecular structure of compound (I), showing the atom labelling. Displacement ellipsoids are drawn at the 30% probability level.

The mol­ecular structure of the title isatin derivative, (I), is illustrated in Fig. 1 ▸. It crystallized in the ortho­rhom­bic space group *P*2_1_2_1_2_1_ with an absolute structure parameter of 0.015 (8). The bond lengths and angles of the isatin moiety are comparable with those reported for similar *N*-substituted isatin derivatives (Qachchachi *et al.*, 2016*a* ▸,*b* ▸).

In compound (I), the isatin ring system is almost planar, with an r.m.s. deviation of the fitted atoms C1–C8/N1/O1/O2 of 0.026 Å. The sum of the bond angles around atom N1 is *ca* 360°, indicating little evidence for the presence of an *sp* ^3^ lone pair.

## Supra­molecular features   

**Figure 2:**
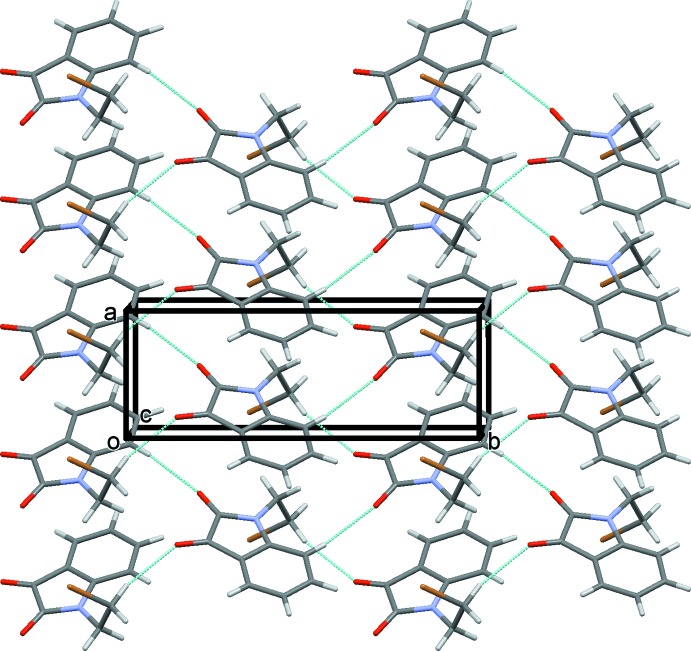
A view along the *c* axis of the crystal packing of compound (I). The hydrogen bonds are shown as dashed lines (see Table 1 ▸) and, for clarity, only H atoms H2 and H10*A* have been included.

In the crystal of (I), mol­ecules are linked by C—H⋯O hydrogen bonds, *viz* C2—H2⋯O1 and C10—H10*A*⋯O2 (Table 1 ▸), which individually form *C*(6) and *C*(7) chains, respectively. Together they form layers parallel to the *ab* plane and enclose 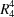
(24) loops (Table 1 ▸ and Fig. 2 ▸). An analysis of the crystal packing of (I) indicated that no further significant inter­molecular inter­actions were present (*PLATON*; Spek, 2009 ▸).

## Database survey   

**Figure 3:**
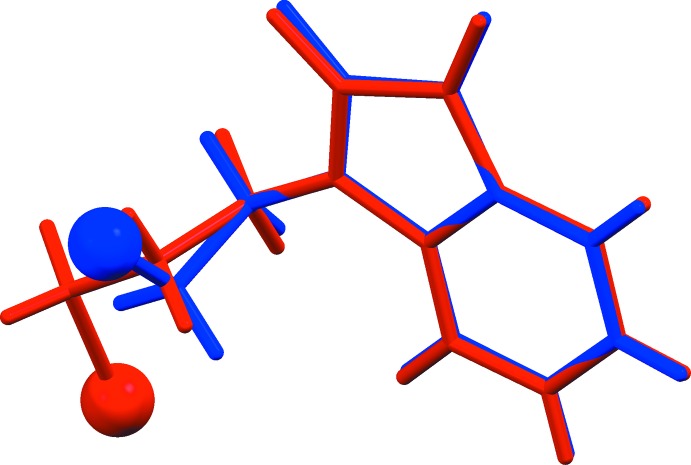
The structural fit of compound (I) and 1-(3-bromo­prop­yl)-1*H*-indole-2,3-dione (AKOBIN; Qachchachi *et al.*, 2016*a* ▸); mol­ecules are shown in blue and red, respectively.

A search of the Cambridge Structural Database (Version 5.37, update May 2016; Groom *et al.*, 2016 ▸) for *N*-substituted isatin derivatives yielded 58 hits. These include five reports of the structure of isatin itself and four reports of the structure of *N*-methyl­isatin. 13 of the structures involve an alkyl chain of two or more C atoms. The compound most similar to the title compound is 1-(3-bromo­prop­yl)-1*H*-indole-2,3-dione (AKO­BIN), whose structure was published very recently (Qachchachi *et al.*, 2016*a* ▸). A view of the structural overlap of this compound with that of compound (I) is shown in Fig. 3 ▸.

## Geometry optimization   

**Figure 4:**
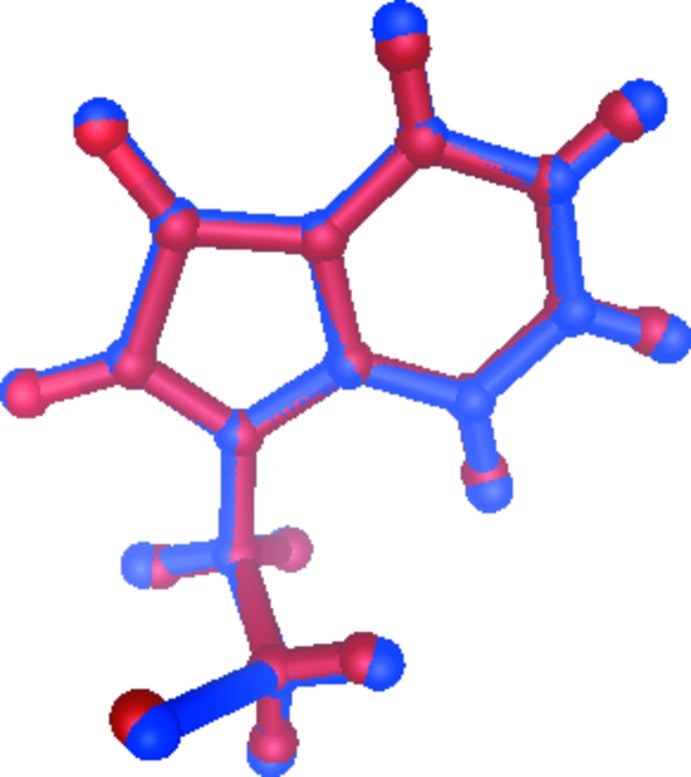
Superimposed fit of the mol­ecule of compound (I) in the crystalline state (red) and after energy minimization (blue).

The geometry optimization of compound (I) was performed using the density functional theory (DFT) method with a 6-311++G** basis set. The crystal structure in the solid state was used as the starting structure for the calculations. The DFT calculations are performed with the *GAUSSIAN09* program package (Frisch *et al.*, 2013 ▸). The resulting geometrical parameters are compared with those obtained from an X-ray crystallography study. A superimposed analysis of (I) with its optimized structure gives an r.m.s. deviation of 0.068 Å (Fig. 4 ▸). This indicates a twist leading to further separation between the isatin moiety and the benzene ring. Also, this suggests that the crystal packing could be influenced by the collective effect of the inter­molecular inter­actions. To probe further, structure-based theoretical parameters, *viz.* HOMO and LUMO energy levels, total energy and dipole moment, were calculated and found to be −6.860 eV, −3.091 eV, −86134.81 eV and 7.2176 Debye, respectively. As a further structure-based test, semi-empirical mol­ecular orbital calculations are carried out using the PM7 method in *MOPAC2012* (Stewart, 2012 ▸; Maia *et al.*, 2012 ▸). The PM7 method gave the HOMO and LUMO energy levels, total energy and dipole moment as −9.276 eV, −1.271 eV, −2334.96 eV and 5.8952 Debye, respectively. Also, the superimposed analysis of the X-ray structure with the isolated mol­ecule in the gas phase by the PM7 method gave an r.m.s. deviation of 0.211 Å. Further, the N1—C8 and N1—C1 (X-ray: 1.367 Å; DFT: 1.392 Å; PM7: 1.424 Å) bond lengths increased, while the bond angles O2—C7—C6 (X-ray: 131.3°; DFT: 130.8°; PM7: 131.2°) and O1—C8—N1 (X-ray: 127.4°; DFT: 126.8°; PM7: 123.8°) decreased. These confirm the influence of the packing inter­actions in the solid state of the mol­ecule. The relative conformation about the bond joining the isatin and bromo­ethyl­ene moieties of (I) is defined by the N1—C9—C10—Br1 torsion angle of 62.0 (5)°. This indicates that the conformation of the mol­ecule is (+)-synclinal.

## Hirshfeld surface analysis   

**Figure 5:**
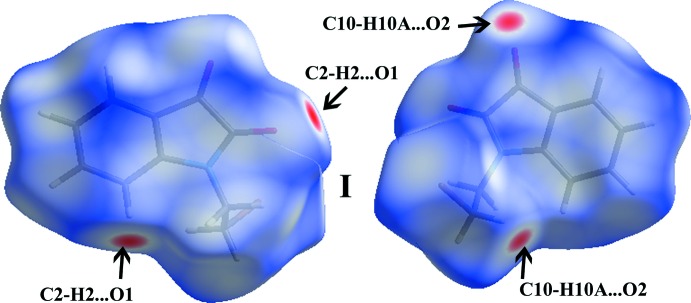
Views of the Hirshfeld surfaces mapped with *d*_norm_ in two different orientations for compound (I). The represented inter­actions are labelled (see Table 1 ▸).

A detailed Hirshfeld surface analysis is useful for identifing the various inter­molecular inter­actions and inter­molecular contacts present in crystal structures, with the aid of decomposed two-dimensional fingerprint plots. The Hirshfeld surface (HS) and the two-dimensional fingerprint plots were generated based on the *d*_i_ and *d*_e_ distances using *Crystal Explorer* (Wolff *et al.*, 2012 ▸); *d*_i_ is the distance from the nearest atom inside the surface, while *d*_e_ is the distance from the HS to the nearest atom outside the surface. This analysis identified the various inter­molecular contacts (O—H, H—H, C—H, C—C and H—Br) and their relative contributions in the crystal structure. The bond lengths (C—H = 1.083 Å, N—H = 1.009 Å and O—H = 0.983 Å) were adjusted to typical neutron diffraction values before the HS calculation (Venkatesan *et al.*, 2015 ▸, 2016*a* ▸,*b* ▸). In Hirshfeld surface diagrams, the contacts with distances shorter than the sum of the van der Waals radii are indicated as red and the contacts with distances longer than the van der Waals radii are represented as blue, whereas the contacts with distances equal to the sum of the van der Waals radii are indicated as white. The HS area of compound (I) is shown in Fig. 5 ▸, and the respective points of inter­molecular inter­actions are labelled.

**Figure 6:**
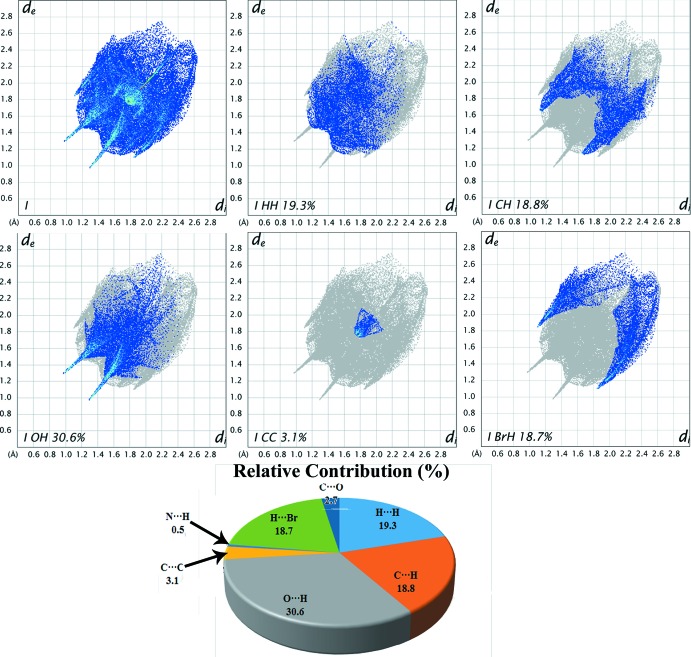
Decomposed two-dimensional fingerprint plots for compound (I). Various close contacts and their relative contributions are indicated.

Two-dimensional fingerprint plots are used to qu­antify and visualize the inter­molecular inter­actions present in the crystal structure and the same for the title compound is shown in Fig. 6 ▸. The result suggests that the share of inter­molecular H⋯H contacts in (I) is about 19.3%. The low percentage could be attributed to the presence of the Br atom in the bromo­ethyl­ene group, which makes *ca* 18.7% contacts with H atoms (Br⋯H). The next significant inter­molecular contacts observed in the structure, *i.e.* O⋯H, C⋯H and C⋯C, have relative contributions of 30.6, 18.8 and 3.1%, respectively.

## Synthesis and crystallization   

To a solution of 1-{2-[(2-bromo­eth­yl)amino]­phen­yl}ethanone (1 equivalent) in DMSO were added I_2_ (0.1 equivalents) and TBHP (1 equivalent, 70% in H_2_O) at ambient temperature, and the mixture was heated to 353 K. The progress of the reaction was monitored by thin-layer chromatography. Upon completion, the reaction mixture was allowed to cool to ambient temperature and was quenched with aqueous sodium thio­sulfate and ethyl acetate. The organic phase was separated, dried over Na_2_SO_4_, filtered and concentrated. The crude product was purified by silica-gel column chromatography using hexa­ne–ethyl acetate (9:1 *v*/*v*) as eluent. The title compound was obtained as a red solid (yield: 71%, 74.5 mg; m.p. 404–406 K). It was dissolved in a mixture of hexa­ne–ethyl acetate (9:1 *v*/*v*) and left to slowly evaporate at room temperature, yielding brown block-like crystals after a period of 3 d.

## Refinement   

Crystal data, data collection and structure refinement details are summarized in Table 2 ▸. C-bound H atoms were included in calculated positions and treated as riding, with C—H = 0.93–0.97 Å and *U*_iso_(H) = 1.2*U*_eq_(C).

## Supplementary Material

Crystal structure: contains datablock(s) I, Global. DOI: 10.1107/S2056989016015760/su5327sup1.cif

Structure factors: contains datablock(s) I. DOI: 10.1107/S2056989016015760/su5327Isup2.hkl

Supporting information file. DOI: 10.1107/S2056989016015760/su5327Isup3.cml

CCDC reference: 1508554

Additional supporting information: crystallographic information; 3D view; checkCIF report

## Figures and Tables

**Table 1:** Hydrogen-bond geometry (Å, °)

*D*—H⋯*A*	*D*—H	H⋯*A*	*D*⋯*A*	*D*—H⋯*A*
C2—H2⋯O1^i^	0.93	2.41	3.286 (6)	156
C10—H10*A*⋯O2^ii^	0.97	2.42	3.309 (6)	151

Symmetry codes: (i) 
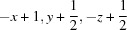
; (ii) 
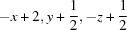
.

**Table 2:** Experimental details

Crystal data	
Chemical formula	C_10_H_8_BrNO_2_
*M*_r_	254.08
Crystal system, space group	Orthorhombic, *P*2_1_2_1_2_1_
Temperature (K)	293
*a*, *b*, *c* (Å)	4.6834 (2), 12.9567 (7), 16.1130 (8)
*V* (Å^3^)	977.76 (8)
*Z*	4
Radiation type	Mo *K*α
μ (mm^−1^)	4.18
Crystal size (mm)	0.25 × 0.20 × 0.20

Data collection	
Diffractometer	Bruker Kappa APEXII CCD
Absorption correction	Multi-scan (*SADABS*; Bruker, 2004 ▸)
*T*_min_, *T*_max_	0.419, 0.498
No. of measured, independent and observed [*I* > 2σ(*I*)] reflections	8226, 3150, 1663
*R*_int_	0.037
(sin θ/λ)_max_ (Å^−1^)	0.762

Refinement	
*R*[*F* ^2^ > 2σ(*F* ^2^)], *wR*(*F* ^2^), *S*	0.055, 0.084, 1.02
No. of reflections	3150
No. of parameters	127
H-atom treatment	H-atom parameters constrained
Δρ_max_, Δρ_min_ (e Å^−3^)	0.70, −0.59
Absolute structure	Flack *x* determined using 503 quotients [(*I* ^+^)−(*I* ^−^)]/[(*I* ^+^)+(*I* ^−^)] (Parsons *et al.*, 2013 ▸)
Absolute structure parameter	0.015 (8)

Computer programs: *APEX2*, *SAINT* and *XPREP* (Bruker, 2004 ▸), *SHELXS97* (Sheldrick, 2008 ▸), *SHELXL2014* (Sheldrick, 2015 ▸), *QMOL* (Gans & Shalloway, 2001 ▸), *Mercury* (Macrae *et al.*, 2008 ▸), *WinGX* (Farrugia, 2012 ▸) and *PLATON* (Spek, 2009 ▸).

## supplementary crystallographic information

### Crystal data

**Table:**

C_10_H_8_BrNO_2_	*D*_x_ = 1.726 Mg m^−^^3^
*M**_r_* = 254.08	Melting point: 406 K
Orthorhombic, *P*2_1_2_1_2_1_	Mo *K*α radiation, λ = 0.71073 Å
*a* = 4.6834 (2) Å	Cell parameters from 2844 reflections
*b* = 12.9567 (7) Å	θ = 2.5–26.7°
*c* = 16.1130 (8) Å	µ = 4.18 mm^−^^1^
*V* = 977.76 (8) Å^3^	*T* = 293 K
*Z* = 4	Block, brown
*F*(000) = 504	0.25 × 0.20 × 0.20 mm

### Data collection

**Table:**

Bruker Kappa APEXII CCD diffractometer	1663 reflections with *I* > 2σ(*I*)
Radiation source: fine-focus sealed tube	*R*_int_ = 0.037
ω and φ scan	θ_max_ = 32.8°, θ_min_ = 2.0°
Absorption correction: multi-scan (SADABS; Bruker, 2004)	*h* = −7→6
*T*_min_ = 0.419, *T*_max_ = 0.498	*k* = −19→17
8226 measured reflections	*l* = −22→18
3150 independent reflections	

### Refinement

**Table:**

Refinement on *F*^2^	Secondary atom site location: difference Fourier map
Least-squares matrix: full	Hydrogen site location: inferred from neighbouring sites
*R*[*F*^2^ > 2σ(*F*^2^)] = 0.055	H-atom parameters constrained
*wR*(*F*^2^) = 0.084	*w* = 1/[σ^2^(*F*_o_^2^) + (0.0167*P*)^2^ + 0.3638*P*] where *P* = (*F*_o_^2^ + 2*F*_c_^2^)/3
*S* = 1.02	(Δ/σ)_max_ < 0.001
3150 reflections	Δρ_max_ = 0.70 e Å^−^^3^
127 parameters	Δρ_min_ = −0.59 e Å^−^^3^
0 restraints	Absolute structure: Flack *x* determined using 503 quotients [(I+)-(I-)]/[(I+)+(I-)] (Parsons *et al.*, 2013)
Primary atom site location: structure-invariant direct methods	Absolute structure parameter: 0.015 (8)

### Special details

**Table:**

Geometry. All esds (except the esd in the dihedral angle between two l.s. planes) are estimated using the full covariance matrix. The cell esds are taken into account individually in the estimation of esds in distances, angles and torsion angles; correlations between esds in cell parameters are only used when they are defined by crystal symmetry. An approximate (isotropic) treatment of cell esds is used for estimating esds involving l.s. planes.

### Fractional atomic coordinates and isotropic or equivalent isotropic displacement parameters (Å^2^)

**Table:**

	*x*	*y*	*z*	*U*_iso_*/*U*_eq_	
Br1	0.83217 (12)	0.83821 (4)	0.06227 (4)	0.0609 (2)	
O1	0.4527 (9)	0.7005 (3)	0.2349 (3)	0.0719 (13)	
O2	0.8447 (9)	0.6409 (2)	0.3684 (2)	0.0688 (10)	
N1	0.6516 (8)	0.8607 (2)	0.2584 (2)	0.0378 (8)	
C1	0.8490 (10)	0.9005 (3)	0.3156 (3)	0.0326 (9)	
C2	0.9288 (9)	1.0020 (3)	0.3268 (3)	0.0429 (12)	
H2	0.8520	1.0548	0.2947	0.051*	
C3	1.1289 (12)	1.0218 (4)	0.3883 (3)	0.0538 (14)	
H3	1.1896	1.0894	0.3966	0.065*	
C4	1.2414 (10)	0.9446 (4)	0.4377 (4)	0.0572 (14)	
H4	1.3755	0.9606	0.4783	0.069*	
C5	1.1551 (10)	0.8445 (4)	0.4267 (3)	0.0503 (11)	
H5	1.2276	0.7920	0.4599	0.060*	
C6	0.9592 (9)	0.8233 (4)	0.3656 (3)	0.0362 (11)	
C7	0.8222 (12)	0.7276 (3)	0.3414 (3)	0.0447 (12)	
C8	0.6167 (11)	0.7569 (4)	0.2703 (3)	0.0458 (13)	
C9	0.4985 (10)	0.9198 (4)	0.1964 (3)	0.0464 (12)	
H9A	0.4179	0.9805	0.2227	0.056*	
H9B	0.3412	0.8784	0.1757	0.056*	
C10	0.6765 (12)	0.9533 (3)	0.1248 (3)	0.0499 (12)	
H10A	0.8325	0.9956	0.1451	0.060*	
H10B	0.5612	0.9955	0.0881	0.060*	

### Atomic displacement parameters (Å^2^)

**Table:**

	*U*^11^	*U*^22^	*U*^33^	*U*^12^	*U*^13^	*U*^23^
Br1	0.0714 (3)	0.0570 (3)	0.0543 (3)	−0.0008 (3)	0.0059 (3)	−0.0069 (3)
O1	0.090 (3)	0.055 (2)	0.071 (3)	−0.036 (2)	−0.003 (2)	−0.012 (2)
O2	0.102 (3)	0.0323 (18)	0.072 (3)	0.005 (2)	0.013 (3)	0.0138 (17)
N1	0.042 (2)	0.035 (2)	0.036 (2)	−0.0055 (19)	−0.001 (2)	0.0022 (16)
C1	0.038 (2)	0.032 (2)	0.028 (2)	−0.001 (2)	0.007 (2)	−0.0028 (18)
C2	0.057 (3)	0.029 (2)	0.043 (3)	−0.001 (2)	0.006 (2)	0.000 (2)
C3	0.069 (4)	0.045 (3)	0.047 (3)	−0.016 (3)	0.011 (3)	−0.015 (3)
C4	0.060 (3)	0.078 (4)	0.034 (3)	−0.011 (2)	0.003 (3)	−0.007 (3)
C5	0.053 (2)	0.063 (3)	0.036 (3)	0.006 (3)	0.004 (3)	0.008 (3)
C6	0.045 (2)	0.038 (3)	0.026 (3)	0.002 (2)	0.006 (2)	−0.001 (2)
C7	0.060 (3)	0.032 (2)	0.043 (3)	0.003 (3)	0.017 (3)	0.002 (2)
C8	0.058 (3)	0.038 (3)	0.041 (3)	−0.011 (3)	0.010 (3)	−0.005 (2)
C9	0.041 (3)	0.053 (3)	0.045 (3)	0.004 (2)	−0.004 (3)	0.000 (3)
C10	0.061 (3)	0.040 (2)	0.048 (3)	0.005 (3)	−0.005 (3)	0.005 (2)

### Geometric parameters (Å, º)

**Table:**

Br1—C10	1.942 (5)	C4—C5	1.370 (7)
O1—C8	1.204 (6)	C4—H4	0.9300
O2—C7	1.210 (5)	C5—C6	1.373 (6)
N1—C8	1.367 (5)	C5—H5	0.9300
N1—C1	1.404 (6)	C6—C7	1.449 (6)
N1—C9	1.449 (6)	C7—C8	1.544 (7)
C1—C2	1.380 (6)	C9—C10	1.488 (7)
C1—C6	1.384 (6)	C9—H9A	0.9700
C2—C3	1.388 (7)	C9—H9B	0.9700
C2—H2	0.9300	C10—H10A	0.9700
C3—C4	1.382 (7)	C10—H10B	0.9700
C3—H3	0.9300		
			
C8—N1—C1	110.4 (4)	C1—C6—C7	107.2 (4)
C8—N1—C9	123.9 (4)	O2—C7—C6	131.3 (5)
C1—N1—C9	125.7 (4)	O2—C7—C8	123.3 (5)
C2—C1—C6	120.7 (4)	C6—C7—C8	105.4 (4)
C2—C1—N1	128.0 (4)	O1—C8—N1	127.4 (5)
C6—C1—N1	111.2 (4)	O1—C8—C7	126.9 (5)
C1—C2—C3	116.9 (4)	N1—C8—C7	105.7 (4)
C1—C2—H2	121.5	N1—C9—C10	114.3 (4)
C3—C2—H2	121.5	N1—C9—H9A	108.7
C4—C3—C2	122.3 (5)	C10—C9—H9A	108.7
C4—C3—H3	118.8	N1—C9—H9B	108.7
C2—C3—H3	118.8	C10—C9—H9B	108.7
C5—C4—C3	119.9 (5)	H9A—C9—H9B	107.6
C5—C4—H4	120.1	C9—C10—Br1	112.9 (3)
C3—C4—H4	120.1	C9—C10—H10A	109.0
C4—C5—C6	118.6 (5)	Br1—C10—H10A	109.0
C4—C5—H5	120.7	C9—C10—H10B	109.0
C6—C5—H5	120.7	Br1—C10—H10B	109.0
C5—C6—C1	121.5 (4)	H10A—C10—H10B	107.8
C5—C6—C7	131.3 (5)		
			
C8—N1—C1—C2	−175.9 (5)	C5—C6—C7—O2	−0.3 (9)
C9—N1—C1—C2	2.7 (7)	C1—C6—C7—O2	−178.2 (6)
C8—N1—C1—C6	2.1 (5)	C5—C6—C7—C8	178.1 (5)
C9—N1—C1—C6	−179.4 (4)	C1—C6—C7—C8	0.2 (5)
C6—C1—C2—C3	1.9 (7)	C1—N1—C8—O1	175.8 (5)
N1—C1—C2—C3	179.7 (4)	C9—N1—C8—O1	−2.8 (8)
C1—C2—C3—C4	−1.2 (7)	C1—N1—C8—C7	−1.8 (5)
C2—C3—C4—C5	−0.1 (8)	C9—N1—C8—C7	179.6 (4)
C3—C4—C5—C6	0.7 (8)	O2—C7—C8—O1	2.0 (8)
C4—C5—C6—C1	0.0 (7)	C6—C7—C8—O1	−176.6 (5)
C4—C5—C6—C7	−177.6 (5)	O2—C7—C8—N1	179.6 (5)
C2—C1—C6—C5	−1.4 (7)	C6—C7—C8—N1	1.0 (5)
N1—C1—C6—C5	−179.5 (4)	C8—N1—C9—C10	−107.9 (5)
C2—C1—C6—C7	176.8 (4)	C1—N1—C9—C10	73.7 (6)
N1—C1—C6—C7	−1.3 (5)	N1—C9—C10—Br1	62.0 (5)

### Hydrogen-bond geometry (Å, º)

**Table:**

*D*—H···*A*	*D*—H	H···*A*	*D*···*A*	*D*—H···*A*
C2—H2···O1^i^	0.93	2.41	3.286 (6)	156
C10—H10*A*···O2^ii^	0.97	2.42	3.309 (6)	151

Symmetry codes: (i) −*x*+1, *y*+1/2, −*z*+1/2; (ii) −*x*+2, *y*+1/2, −*z*+1/2.
